# Supplementary material for: Efficient encoding of large antigenic spaces by epitope prioritization with Dolphyn
Source: Nat Commun. 2024 Feb 21;15:1577. doi: 10.1038/s41467-024-45601-8 (PMC10881494; doi:10.1038/s41467-024-45601-8)
Supplement: Supplementary file 1 — Supplementary Information [file 41467_2024_45601_MOESM1_ESM.pdf]

Supplementary Information for

Efficient encoding of large antigenic spaces by epitope prioritization with Dolphyn

Figure S1. Dolphyn algorithm in detail

Dolphyn predicts for each 15 amino acid sub-peptide (15-mer) of a protein sequence whether it contains an epitope using our random forest model described in the manuscript. The Figure gives a depiction of what happens in the algorithm. The corresponding pseudo code description can be found in Box1 in the main manuscript.

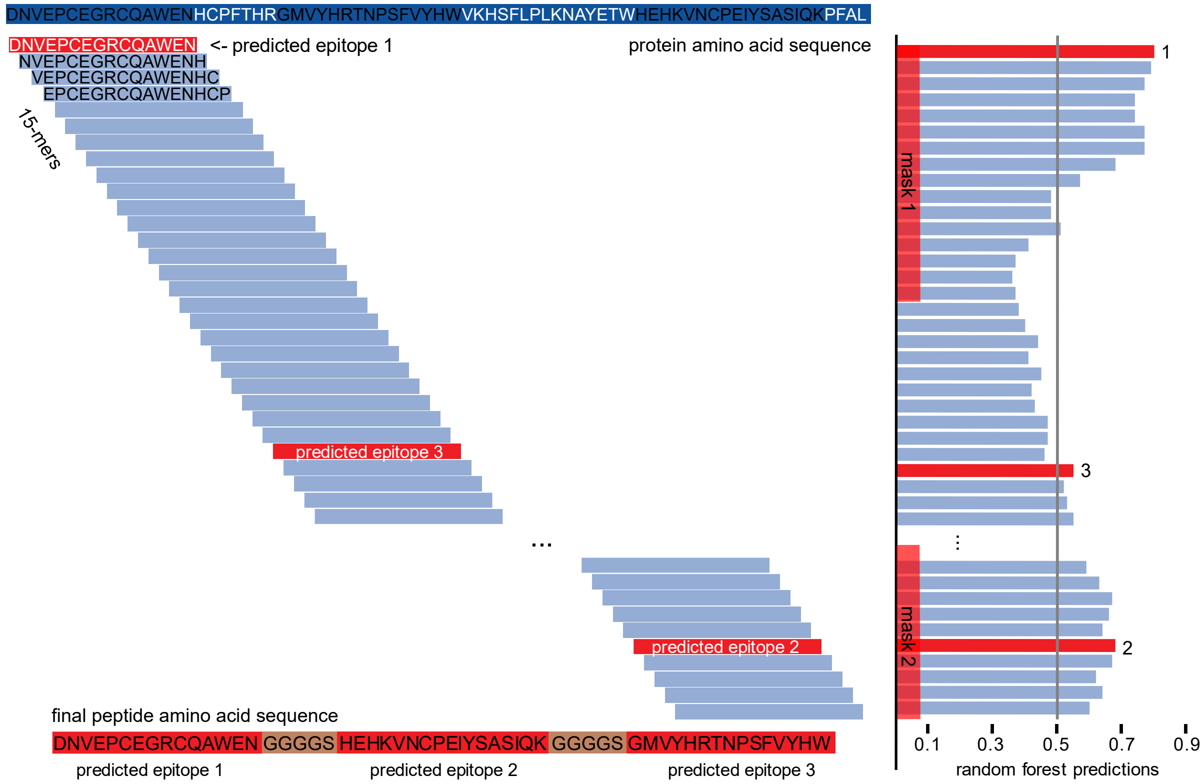

Figure S1 - Dolphyn algorithm in detail. A prediction profile is created as displayed on the right. The 15-mer with the highest probability is chosen first and all 15-mer predictions that overlap with this first 15-mer are masked out before selecting the next highest 15-mer prediction. The masking and selecting is repeated until there are no more 15-mers with prediction values greater than the cut-off (default 0.5). For creating the stitched peptides, the highest probability 15-mers are chosen in a number that is divisible by three. For example, if a protein sequence contains seven potential epitopes, six of them are used for stitching onto two peptides that represent this protein.

Figure S2. Linker sequences

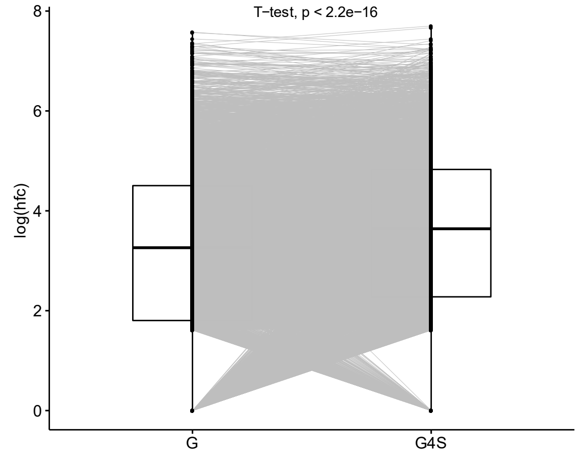

Figure S2 – Linker sequences. Paired t-test of 76 triplet peptides in 176 individuals (6466 data points, excluded both-zero datapoints) separated by G or GGGGS linker. Horizontal lines in boxplot indicate the median, and lower and upper value of the box the interquartile ranges. The entire data point range is displayed. | Source data are provided as a Source Data file.

In Dolphyn's "stitching" step, three 15 amino acid long peptides are combined onto one peptide and separated via a flexible and inert linker sequence GGGGS. We evaluated two different linkers, a single Glycine (G) or four Glycine and one Serine (G4S = GGGGS). We found that the G4S linker allows a higher reactivity value (fold change versus mock IP) for the same peptides.

A validation library was created from the Public Epitope Data Set (PEDS) for evaluating the stitching approach and two different linker sequences between the epitopes. Having ground truth for these 15 amino acid long peptides, we include the 48 most reactive ones, i.e., the ones reactive in the highest number of tested samples, into this validation library. Further, we included 96 "non-reactive" peptides in the library which are those that have high bead counts in the quality control samples, but no reactivity in the tested patients. Any sequence shorter than 56 amino acids was filled up with three stop codons and a random sequence of DNA nucleotides. Then, we stitched the 48 reactive peptides onto 16 tiles with three epitopes, each and 96 non-reactive one onto 32 tiles. Next, we combined reactive and non-reactive peptides randomly with three peptides per tile. All stitched peptides are synthesized twice, with the two different linkers. This library has a total of 336 peptide tiles.

Figure S3. Protein discovery power of library peptides

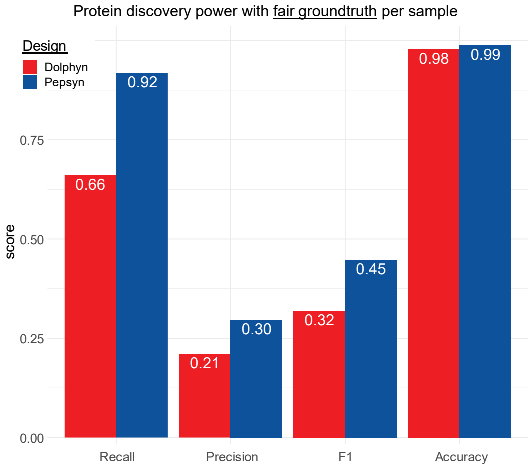

Figure 5D in the manuscript shows the performance metrics recall, precision, F1 and accuracy for Dolphyn-library and Pepsyn-library peptides. All peptide-sample pairs are compared to the ground-truth. The ground-truth in Figure 5D is defined from the Pepsyn library. A positive hit is used to refer to any peptide in a protein reactive in an individual. The performance metrics then are calculated for all peptides in the library (which is why Pepsyn's recall is not 1).

Figure S3 - Protein discovery power of library peptides The ground-truth is defined if any peptide of both libraries, Pepsyn or Dolphyn, in a protein displays reactivity in an individual. Performance metrics are shown for both Pepsyn and Dolphyn peptides in recovering protein by sample reactivities. | Source data are provided as a Source Data file.

Figure S4. All reactive peptides in an individual

Figure 4A in the manuscript shows for four individuals all peptide quartets (three 15-mers and their stitched version) where two or more peptides show reactivity. Here, exemplary for individual 2, all reactive peptides are shown in the same format. Not all reactive individual peptides are also reactive in the stitched version, and there are stitched peptides that could not be confirmed by any individual epitope.

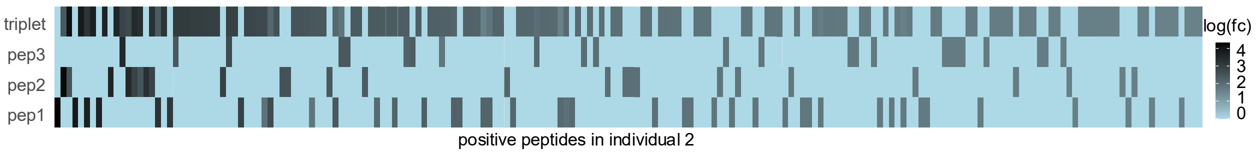

Figure S4 - Reactive peptides in an individual. Relationship between reactivities of stitched Dolphyn peptides and unstitched predicted epitope peptides. All peptide quartets (three 15-mers and their stitched version) are shown. Peptide sets are ordered by highest to lowest reactivity from left to right. | Source data are provided as a Source Data file.

Figure S5. Validation of Dolphyn peptides

The predicted epitope 15-mers were synthesized in a separate library to the Dolphyn stitched peptide library. In a paired t-test (1,882 degrees of freedom, leading to a standard Gaussian null distribution in essence), the null hypothesis of no difference in means in reactivity between the libraries was rejected with a p-value of 0.0015 (Fig S5A). As shown in Fig S4, not all reactive individual peptides are also reactive in the stitched version, or the other way round.

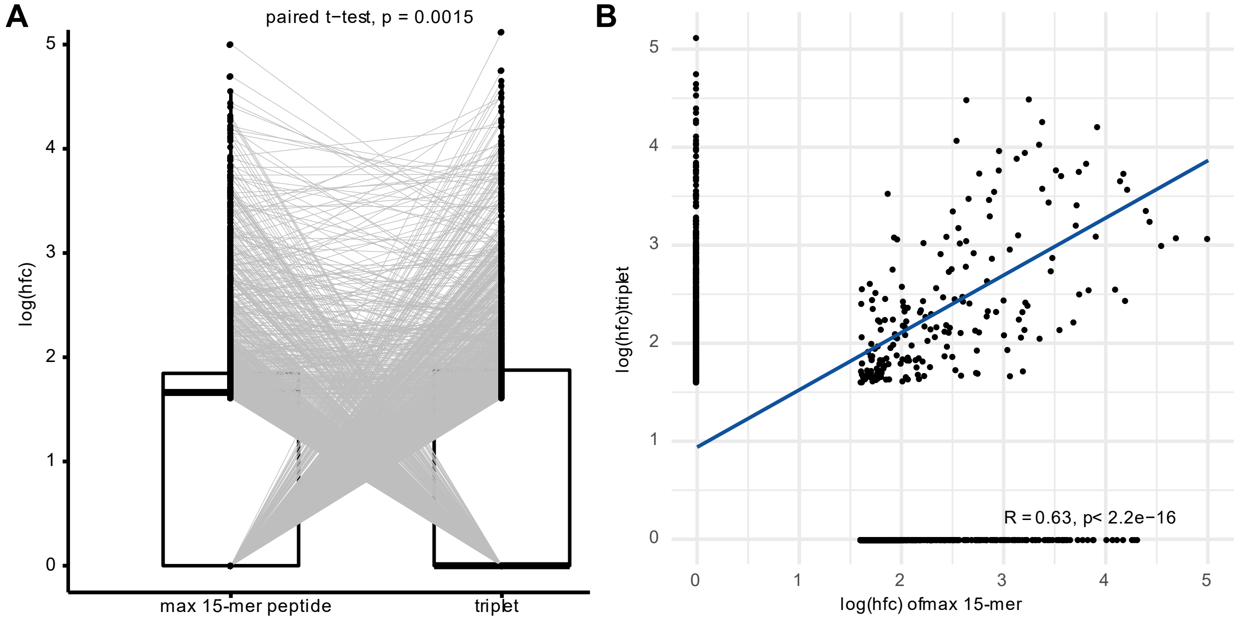

*Figure S5 - Validation of Dolphyn peptides. The Dolphyn stitched peptide library and the individual 15-mers (predicted epitopes) were synthesized in separate libraries. **A** Paired t-test of 1468 peptides in each library and their reactivity in 4 individuals (1883 datapoints, excluded all-zero datapoints, differences in means = 0.14). Horizontal bars in boxplots indicate the median, and lower and upper values of the box are interquartile ranges. The entire data point range is displayed. **B** Correlation of 1468 peptide reactivities in 4 individuals (1883 datapoints). Linear regression line and Pearson correlation given for non-zero datapoints. | Source data are provided as a Source Data file.*

Table S1. Cohort for Public Epitope Data Set

439 plasma samples were obtained from the Genital Shedding (GS) Study (Uganda and Zimbabwe; 2001-2009) for the creation of the public epitope dataset (PEDS). Table S3:

**Characteristic**	**Cohort**
Country of origin	Zimbabwe
HIV subtype	C
Number of samples	425 + 14 replica
Number of participants	59
Duration of HIV infection (years)	0.04 to 8.8
Mean samples per participant, (range)	7 (3 - 9)
Female sex, % of participants	100%
Age at seroconversion (years)	19-37

Available on GitHub: Public epitope visualization

The GitHub repository features a PDF which visualizes each wildtype contained in the public epitope dataset individually. Each page contains a graphic, such as the following, where each line is the alanine scan for one sample. In particular, the value on the y-axis in the difference in fold-change from the wildtype fold-change.

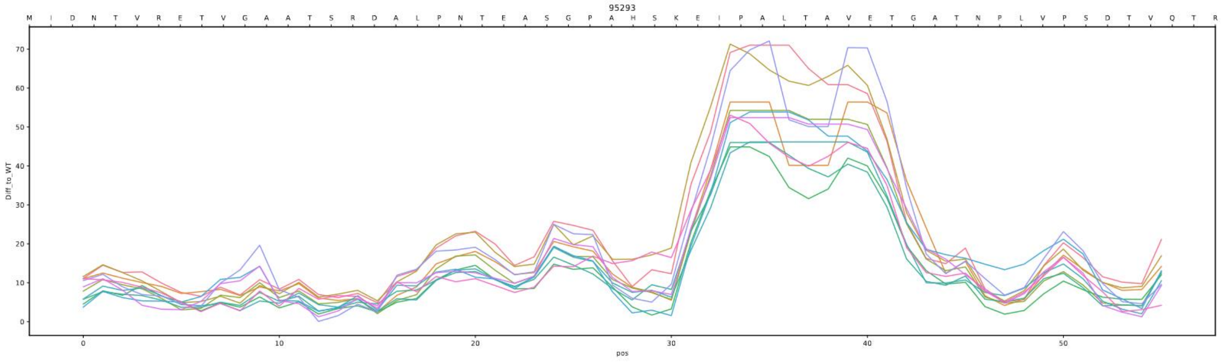

Sample page of file *AlanineScan_DiffToWT.pdf* on https://github.com/kepsi/Dolphyn .
