# Supplementary material for: Efficient encoding of large antigenic spaces by epitope prioritization with Dolphyn
Source: Nat Commun. 2024 Feb 21;15:1577. doi: 10.1038/s41467-024-45601-8 (PMC10881494; doi:10.1038/s41467-024-45601-8)
Supplement: Supplementary file 3 — Reporting Summary [file 41467_2024_45601_MOESM3_ESM.pdf]

nature portfolio

Corresponding author(s): Larman, HB

Last updated by author(s): Dec 29, 2023

Reporting Summary

Nature Portfolio wishes to improve the reproducibility of the work that we publish. This form provides structure for consistency and transparency in reporting. For further information on Nature Portfolio policies, see our Editorial Policies and the Editorial Policy Checklist.

Statistics

For all statistical analyses, confirm that the following items are present in the figure legend, table legend, main text, or Methods section.

n/a	Confirmed	
☐	☒	The exact sample size (n) for each experimental group/condition, given as a discrete number and unit of measurement
☐	☒	A statement on whether measurements were taken from distinct samples or whether the same sample was measured repeatedly
☐	☒	The statistical test(s) used AND whether they are one- or two-sided<br>*Only common tests should be described solely by name; describe more complex techniques in the Methods section.*
☐	☒	A description of all covariates tested
☐	☒	A description of any assumptions or corrections, such as tests of normality and adjustment for multiple comparisons
☐	☒	A full description of the statistical parameters including central tendency (e.g. means) or other basic estimates (e.g. regression coefficient) AND variation (e.g. standard deviation) or associated estimates of uncertainty (e.g. confidence intervals)
☐	☒	For null hypothesis testing, the test statistic (e.g. F, t, r) with confidence intervals, effect sizes, degrees of freedom and P value noted<br>*Give P values as exact values whenever suitable.*
☒	☐	For Bayesian analysis, information on the choice of priors and Markov chain Monte Carlo settings
☐	☒	For hierarchical and complex designs, identification of the appropriate level for tests and full reporting of outcomes
☐	☒	Estimates of effect sizes (e.g. Cohen's d, Pearson's r), indicating how they were calculated

Our web collection on statistics for biologists contains articles on many of the points above.

Software and code

Policy information about availability of computer code

Data collection	Sequencing data was collected using Illumina Real-Time Analysis software on the sequencing instrument. We utilized exact matching implemented in R v 4.2.2 to map sequencing reads to PhIP-Seq library members producing counts data.
Data analysis	Data analysis was performed in R v 3.6.3 and Python 3.8. Enrichment of antibody reactivities was determined via the edgeR package (v.3.32.0). R packages used for analysis include reshape2 (v 1.4.4), dplyr (v 1.0.9) and ARscore (v 0.2.0). Python packages used include pandas (v 1.2.4), numpy (v 1.19.5) and sklearn (v 0.24.2). Plots were generated with the ggplot2 R package (v 3.3.6) and heatmap.2 of the gplots (v 3.1.3) R package and matplotlib (v 3.4.2) Python package. The Dolphyn package was developed as part of this manuscript and is available in the GitHub repository (folder dolphyn) alongside the scripts deriving the results (folder Manuscript Analyses) on https://github.com/kepsi/Dolphyn.

For manuscripts utilizing custom algorithms or software that are central to the research but not yet described in published literature, software must be made available to editors and reviewers. We strongly encourage code deposition in a community repository (e.g. GitHub). See the Nature Portfolio guidelines for submitting code & software for further information.

Data

Policy information about availability of data

All manuscripts must include a data availability statement. This statement should provide the following information, where applicable:

- Accession codes, unique identifiers, or web links for publicly available datasets
- A description of any restrictions on data availability
- For clinical datasets or third party data, please ensure that the statement adheres to our policy

All data was made publicly available on Zenodo: https://doi.org/10.5281/zenodo.7979556
A GitHub repository (https://github.com/kepsi/Dolphyn) contains the processed PhIP-seq and source data files.

Research involving human participants, their data, or biological material

Policy information about studies with human participants or human data. See also policy information about sex, gender (identity/presentation), and sexual orientation and race, ethnicity and racism.

Reporting on sex and gender	All samples used in this study stem from cohorts originally sampled for other studies (partially published, see manuscript) and re-used for evaluating and validating the novel algorithm developed in the study. Details about sample collection procedures were not provided to the study team.<br>EV cohort: 3 male and 3 female children<br>VRC cohort: The cohort included 446 men, 351 women and 4 "unknown".<br>PEDS cohort: Female only cohort.
Reporting on race, ethnicity, or other socially relevant groupings	All samples used in this study stem from cohorts originally sampled for other studies (partially published, see manuscript) and re-used for evaluating and validating the novel algorithm developed in the study. Reasoning about social composition of the cohorts were not provided to the study team.<br>EV cohort: non-Hispanic white<br>VRC cohort: Of the 801 individuals, 535 are of European genetic ancestry, 194 of African genetic ancestry, 32 of Asian genetic ancestry and 40 belonging to other ancestral groups.<br>PEDS cohort: All participants from Uganda and Zimbabwe.
Population characteristics	EV cohort: 3 Type-1 Diabetes positive and 3 negative individuals, aging 0-7 years<br>VRC cohort: healthy community volunteers in the greater Baltimore/Washington DC area, ages 18 to 64, average age 39<br>PEDS cohort: HIV C infected individuals, aging 19-37 years at seroconversion
Recruitment	All samples used in this study stem from cohorts originally collected for other studies (see manuscript) and re-used for evaluating and validating the novel algorithm developed in the study. Recruitment details were not provided to the study team.
Ethics oversight	EV cohort: The Colorado Multiple Institutional Review Board approved all study protocols. The Diabetes Auto Immunity Study in the Young (DAISY) is a prospective study of children with increased risk for Type 1 Diabetes [NCT03205865].<br>VRC cohort: Vaccine Research Center (VRC)/National Institutes of Allergy and Infectious Diseases (NIAID/NIH protocol "VRC000: Screening Subjects for HIV Vaccine Research Studies" (NCT00031304)<br>PEDS cohort: Institutional Review Board of Johns Hopkins University

Note that full information on the approval of the study protocol must also be provided in the manuscript.

Field-specific reporting

Please select the one below that is the best fit for your research. If you are not sure, read the appropriate sections before making your selection.

☒ Life sciences ☐ Behavioural & social sciences ☐ Ecological, evolutionary & environmental sciences

For a reference copy of the document with all sections, see nature.com/documents/nr-reporting-summary-flat.pdf

Life sciences study design

All studies must disclose on these points even when the disclosure is negative.

Sample size	All available biologic samples from the described cohorts were utilized, except stated otherwise.
Data exclusions	No data was excluded from these analyses.
Replication	PhIP-Seq was performed on a subset of biological samples in duplicate to ensure reproducibility of individual antibody profiles.
Randomization	By design, this study does not include experimental grouping of samples.

Blinding	All experimentation was performed without knowledge of sample groups.

Reporting for specific materials, systems and methods

We require information from authors about some types of materials, experimental systems and methods used in many studies. Here, indicate whether each material, system or method listed is relevant to your study. If you are not sure if a list item applies to your research, read the appropriate section before selecting a response.

Materials & experimental systems

n/a	Involved in the study
☒	☐ Antibodies
☒	☐ Eukaryotic cell lines
☒	☐ Palaeontology and archaeology
☒	☐ Animals and other organisms
☒	☐ Clinical data
☒	☐ Dual use research of concern
☒	☐ Plants

Methods

n/a	Involved in the study
☒	☐ ChIP-seq
☒	☐ Flow cytometry
☒	☐ MRI-based neuroimaging
